# Simulating human foot mechanics during walking based on an anatomically detailed forward dynamic finite element model

**DOI:** 10.1007/s10439-026-03984-3

**Published:** 2026-01-13

**Authors:** Kohta Ito, Yuka Matsumoto, Hiroyuki Seki, Takeo Nagura, Naomichi Ogihara

**Affiliations:** 1 Department of Biological Science, Graduate School of Science, The University of Tokyo, Tokyo, Japan; 2 Graduate School of Human Sciences, The University of Osaka, Suita, Japan; 3 Research Development Center, Saitama Prefectural University, Koshigaya, Japan; 4 Department of Orthopedic Surgery, Tachikawa Hospital, Tachikawa, Japan; 5 Department of Clinical Biomechanics, Keio University School of Medicine, Tokyo, Japan

**Keywords:** Foot biomechanics, Musculoskeletal model, Gait, Ground reaction force, Bipedal locomotion

## Abstract

**Purpose:**

Forward dynamic musculoskeletal simulation is a powerful computational approach for investigating the biomechanics of human locomotion. However, existing models often oversimplify foot anatomy, thereby limiting our understanding of the role of detailed foot morphology in gait mechanics. In this study, we developed an anatomically accurate three-dimensional finite element (FE) model of the human foot to simulate its dynamic behavior during the stance phase of walking using an explicit forward dynamics approach.

**Methods:**

The model incorporated detailed representations of bones, soft tissues, ligaments, and the plantar aponeurosis and was driven by experimentally measured tibial kinematics and estimated muscle forces.

**Results:**

Simulation results were consistent with experimental data on ground reaction forces, plantar pressure distributions, and bone movements, confirming the model’s ability to replicate key aspects of foot–ground interactions during walking. Moreover, the model enabled the estimation of internal forces, stresses, and strains in foot structures that are not directly measurable in vivo, offering new insights into the biomechanics underlying foot pathologies.

**Conclusions:**

This study potentially provides a robust framework for exploring the form–function relationship of the human foot, with applications in evolutionary biology, clinical interventions, and the study of locomotor disorders.

**Supplementary Information:**

The online version contains supplementary material available at 10.1007/s10439-026-03984-3.

## Introduction

Forward dynamic musculoskeletal simulation is a computational technique to calculate muscle-driven skeletal movements through the integration of differential equations governing the dynamics of a musculoskeletal system [40, 58]. Extensive research in forward dynamic simulations has focused on bipedal walking, aiming to understand the complex biomechanics and neurocontrol mechanisms governing human locomotion [e.g., 53, 57, 38, 47, 23, 35, 2, 49, 31, 10, 39, 16]. These simulations also hold significance in predicting alterations in the kinematics, kinetics, and energetics of walking resulting from virtual alterations to the musculoskeletal system. For example, Oku et al. [39] demonstrated using a forward dynamic musculoskeletal simulation that the manipulation of the foot structure from digitigrade to humanlike plantigrade to allow heel contact results in improved cost of transport, suggesting that evolutionary changes in the foot structure were crucial for the acquisition of humanlike efficient bipedal locomotion. Such virtual alterations offer a unique opportunity for exploring the potential impact of anatomical changes or interventions on human gait, thus providing valuable insights into understanding the form–function relationship of the musculoskeletal system and human movement disorders.

However, in these models, it is not feasible to assess how variations in the morphology of small foot bones impact the forces acting on the foot, and thus, the overall dynamics of the body. This limitation arises because the foot is typically represented as either a single rigid body [2, 10, 23, 35, 38, 47, 49, 53, 57] or a two rigid-link model comprising the foot and toe [16, 31, 39]. Such simplified representations are sufficient for reproducing whole-body kinematics and ground reaction forces, but they do not allow a detailed assessment of how local bony or soft tissue variations in foot morphology or internal load-sharing among bones, ligaments, and the plantar aponeurosis influence joint loading within the foot. Several detailed multi-segment foot models have been developed [29, and references therein]. However, most of these models do not represent detailed bone and soft tissue morphologies and have been applied primarily in kinematic and inverse dynamics analyses, rather than being embedded in forward dynamic simulations of walking. There is therefore still a need for anatomically realistic FE foot models that can be coupled with forward dynamic musculoskeletal simulations to bridge external gait mechanics and internal tissue loading.

The objective of this study is to develop and validate an anatomically detailed FE model of the human foot, incorporating explicit three-dimensional (3D) representations of the small tarsal bones, major ligaments, and plantar aponeurosis. This model is designed to estimate foot bone kinematics, internal joint contact forces, and ligament loading under physiologically relevant loading conditions and to provide a basis for future integration with forward dynamic simulations of bipedal walking. While a previous study conducted a two-dimensional dynamic FE analysis of the foot during walking to elucidate its dynamic and functional interaction with the ground [42], more recent efforts have seen the emergence of 3D explicit dynamic FE analyses of the human foot during both walking [1, 33, 55] and running [7]. However, these 3D FE foot models were typically validated primarily against experimentally obtained plantar pressure distributions applied to the foot’s plantar surface, neglecting validation against horizontal (anteroposterior and mediolateral) ground reaction forces and bone kinematics. Consequently, while these studies successfully replicated foot mechanics during walking and running using 3D human foot models, there may exist potential limitations in accurately reproducing foot dynamics. In addition, two of the previous FE gait studies only analyzed a few discrete stance postures quasi-statically [1, 55]. The only explicit dynamic analysis to date (Mo et al. [33]) drove the foot with prescribed kinematics and ground reaction forces rather than muscle-driven gait. Thus, to our knowledge, explicit dynamic FE simulations of the foot under muscle-driven loading throughout the entire stance phase have not yet been reported. A forward dynamic simulation of human walking that uses an anatomically realistic foot model and reproduces realistic kinematics and kinetics continuously during the stance phase of human walking would provide a useful framework for linking musculoskeletal form to function and for investigating human movement disorders.

## Method

### Human foot model

**Fig. 1:**
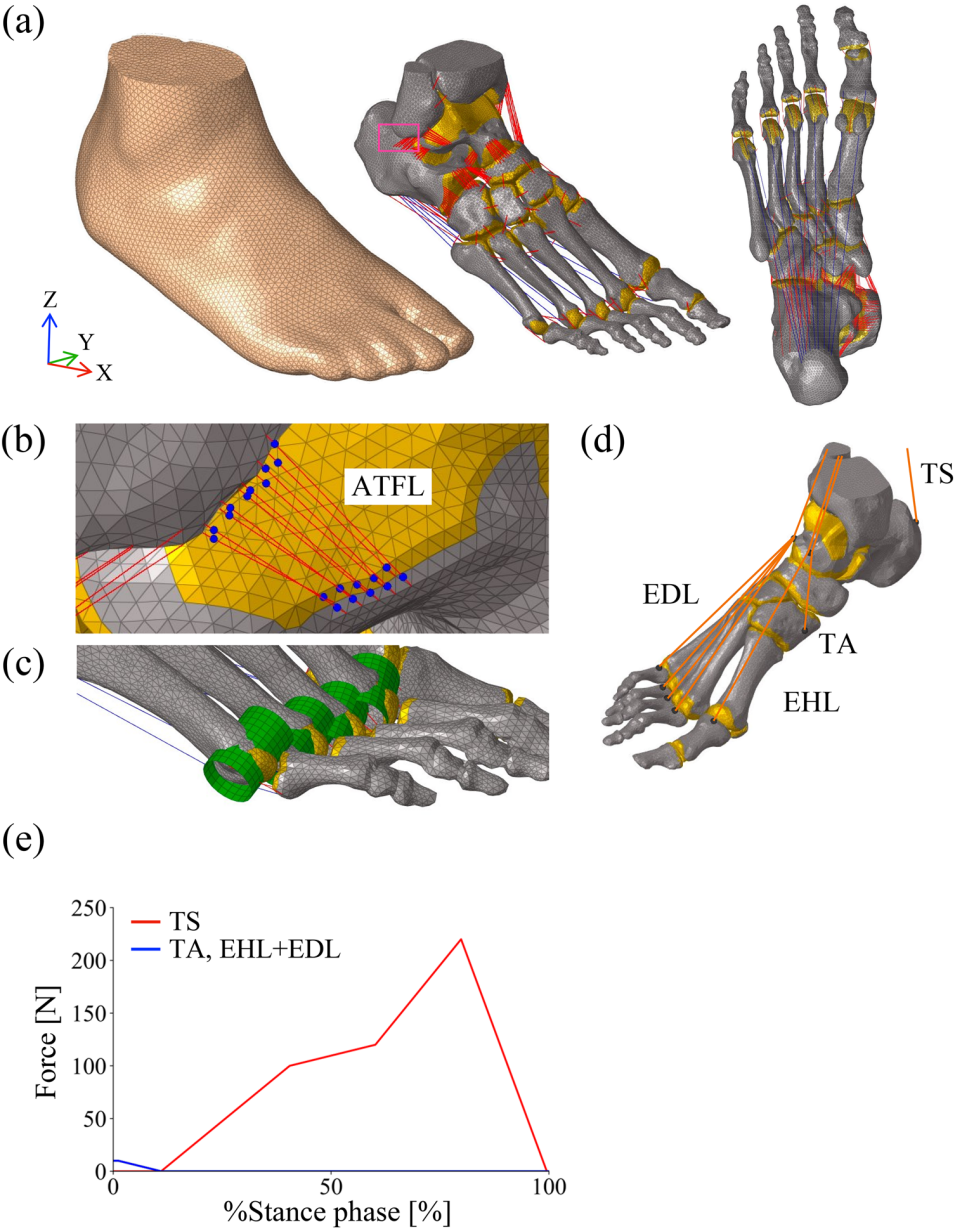
Finite element models of the human foot. (**a**) Skeletal and soft tissue components. Ligaments and the plantar aponeurosis (PA) are shown as red and blue lines, respectively. Cartilages are represented as yellow areas. The dorsal view illustrates the overall configuration of the PA. (**b**) Enlarged view of the anterior talofibular ligament (ATFL). Ligaments are modeled as bundles of an alternating arrangement of tension-only springs and non-penetrating spheres (blue). (**c**) To prevent the penetration of the PA elements during metatarsophalangeal dorsiflexion, the second to fifth metatarsal heads are approximated as cylindrical surfaces (green). (**d**) Four muscles involved in walking, triceps surae (TS), tibialis anterior (TA), extensor digitorum longus (EDL), and extensor hallucis longus (EHL), are modeled as line segments extending from origins to insertions via intermediate points (orange). (**e**) Muscle force profiles applied to replicate physiological loading conditions during the stance phase of walking. Because the numerical values were not available, the graphs were digitized from the published figure [38] and sampled at discrete time points over the stance phase. Linear interpolation between these points was then used to prescribe time-varying muscle forces in the simulation

In the present study, the 3D FE model of the human foot developed in Ito et al. [22] was updated to be anatomically more realistic and accurate (Figure 1a). This model was developed for quiet standing. Because of its simplified ligament representation, the model exhibited unrealistic ligament–bone penetration when applied to walking.

The foot model was constructed based on CT scan data of a healthy male adult (age, 42 years; height, 172 cm; weight, 72 kg; foot length, 25.5 cm) with no history of orthopedic and neuromuscular impairments. The foot model consisted of a total of 23 bones: tibia, fibula, talus, calcaneus, navicular, cuboid, three cuneiforms, five metatarsals, proximal and distal hallucal phalanges, and four fused phalanges of the second to fifth rays, and three sesamoids, all meshed with tetrahedral elements. The bones were represented as an isotropic linear elastic material, and the Young’s modulus, Poisson’s ratio, and density were set to 7300 MPa, 0.3, and 0.0015 g/mm^3^, respectively [8, 42] (Table 1). The encapsulated soft tissues between the outer foot surface and bone surface were also meshed with tetrahedral elements. The encapsulated soft tissue was defined as a hyperelastic Ogden material. The uniaxial nominal stress–stretch (*σ*–*λ*) equation for this material is represented as follows:

1 $$\sigma = - \frac{2C}{\alpha }(\lambda^{\alpha - 1} - \lambda^{ - \alpha /2 - 1} )$$

where *λ* is the deviatoric principal stretch, and *C* and *α* are coefficients. The coefficients, *C* and *α*, density and Poisson’s ratio of which were 0.0102 MPa, 8.04 [52], 0.937 × 10^−3^ g/mm^3^ [42], and 0.475 (nearly incompressible), respectively. The viscous properties of the soft tissue, the relaxation coefficients (g_1_, g_2_) and time constants (τ_1_, τ_2_) were 0.18, 0.12, 0.57, and 6.03, respectively [51]. The outer surface of the foot was modeled as a uniform layer of the skin with a thickness of 1 mm [50] and meshed with three-node triangular shell elements. The material properties of the skin were represented as a hyperelastic Ogden material, and coefficients *C* and *α* were determined to be 0.122 MPa and 18, respectively [18]. The density and Poisson’s ratio of the skin were assumed to be identical to those of the soft tissue.

**Table 1:** Components and material properties of the human foot finite element model

Component	Element type	Material model	Key parameters	Density (g/mm^3^)	Reference
Bones	4-node tetrahedral solids	Linear elastic, isotropic	E = 7300 MPa ν = 0.30	1.5 × 10⁻^3^	[8, 42]
Encapsulated soft tissue	4-node tetrahedral solids	Hyperelastic Ogden + viscoelastic (Prony series)	C = 0.0102 MPa α = 8.04 ν = 0.475 g₁ = 0.18, g₂ = 0.12 τ₁ = 0.57 s, τ₂ = 6.03 s	0.937 × 10⁻^3^	[42, 51, 52]
Skin Thickness = 1.0 mm	3-node triangular shells	Hyperelastic Ogden	C = 0.122 MPa α = 18 ν = 0.475	0.937 × 10⁻^3^	[18, 42, 50]
Articular cartilage Thickness ≈ 0.5-1.0 mm	3-node triangular shells or selected solid elements at joint surfaces	Linear elastic	E = 10 MPa ν = 0.40 Friction: μ = 0	2.0 × 10⁻^3^	[17, 42]
Ligaments	Tension-only springs + 1.0 mm spheres arranged in series	Linear elastic (spring elements)	E = 260 MPa ν = 0.40	1.0 × 10⁻^3^	[7] Supplementary Information
Plantar aponeurosis (PA)	Tension-only springs (10) wrapping over metatarsal heads; first element passing through sesamoids	Linear elastic (spring elements)	E = 412.02 MPa ν = 0.40	1.0 × 10⁻^3^	[5, 28]
Floor / ground contact	Rigid surface	–	Friction: μ = 0.6	–	[8]

Elements corresponding to the articular cavity were manually removed, and thin cartilaginous layers of approximately 0.5 to 1 mm thickness were added on the articular surfaces of the talocrural, talocalcaneal, and calcaneocuboid joints, which were meshed with three-node triangular shell elements. For the other joints, the bone elements corresponding to articular surfaces were selected and assigned as articular cartilage. The Young’s modulus, Poisson’s ratio, and density of the articular cartilage were set to 10 MPa, 0.4, and 0.002 g/mm^3^, respectively [17, 42]. Articular surface-to-surface contact was simulated using a frictionless contact model. The horizontal floor was modeled as a rigid wall, and the contact between the plantar surface of the foot and floor was modeled using a contact model with friction, with the coefficient of static and dynamic friction set to 0.6 [8].

In the foot model outlined in Ito et al. [22], ligaments were depicted as tension-only spring elements linking origin and insertion points according to anatomical atlases [11, 44], resulting in ligament penetration into connecting bones. Moreover, many foot ligaments possess a sheet-like structure with large attachment sites, yet this anatomical feature was not incorporated in the model. In the present model, each ligament is represented as an alternating arrangement of tension-only springs and spheres, 1.0 mm in diameter, forming a connected series spanning from origin to insertion points (Figure 1b). The spheres were not rigidly fixed to the bone surface but interacted with it via a frictionless contact formulation, allowing them to slide over the bone without penetrating it. In this way, the ligament path could wrap smoothly around bony contours while remaining in contact with the underlying surface. To accommodate the relatively large attachment sites, ligaments around the talocrural, talocalcaneal, talonavicular, and calcaneocuboid joints are modeled as bundles of 10 such structures each. The Young’s modulus, Poisson’s ratio, and density of the ligament were 260 MPa, 0.4, and 0.001 g/mm^3^ [7]. The cross-sectional areas of the foot ligaments were determined based on Mkandawire et al. [32], Ito et al. [22], Jotoku et al. [24], Siegler et al. [48], Edama et al. [12–14], Taniguchi et al. [54], Won et al. [56], Sarrafian and Kelikian [43], and Maas et al. [26], as described in the Supplementary Information.

Experimentally determining the slack length of ligaments (and other musculoskeletal soft tissue structures) is extremely challenging; therefore, many studies adopt the tissue length in a defined body posture as the slack length [59]. In the present study, the slack length of each ligament was assumed to be 1.1 times its length in the CT-scanned posture. This is because if the slack length is underestimated, the ligaments remain constantly tensioned, resulting in unrealistically high stiffness and contact forces. However, because the ankle and foot were slightly inverted and adducted during scanning, the lateral hindfoot ligaments (e.g., anterior talofibular, calcaneofibular, lateral talocalcaneal, lateral calcaneocuboid, bifurcate, and posterior talofibular ligaments) were likely stretched relative to their slack length. Accordingly, their slack lengths were defined as their lengths in the CT-scanned posture. Similarly, the plantar ligaments (e.g., plantar calcaneocuboid, plantar calcaneonavicular, plantar cubonavicular, plantar cuneonavicular, plantar cuneocuboid, plantar intercuneiform, plantar tarsometatarsal, plantar metatarsal, short plantar, and long plantar ligaments) were not expected to have been shortened during scanning; therefore, their slack lengths were also defined as their lengths in the CT-scanned posture.

The plantar aponeurosis (PA) was modeled using 10 tension-only spring elements that connected the origin and insertion points, with the first PA element passing through the sesamoids beneath the hallux (Figure 1a). To prevent penetration of the second to fifth PA elements during metatarsophalangeal (MP) joint dorsiflexion, the articular surfaces of the metatarsal heads were approximated as cylindrical surfaces, allowing the PA elements to wrap smoothly around them (Figure 1c). The Young’s modulus of the PA was estimated based on Caravaggi et al. [5], who reported that the tensile force of the PA was approximately 1.5 times the body weight when the strain was 0.05. Assuming a body weight of 70 kg and the cross-sectional area of 50 mm^2^ [28], the value was calculated to be 412.02 MPa. The Poisson’s ratio and density of the PA’s were 0.4 and 0.001 g/mm^3^, respectively. The slack length of the PA was defined as its length in the CT-scanned posture, as the metatarsophalangeal joints were slightly flexed in that posture.

### Forward dynamic simulation

In the present study, the mechanical interactions of the foot with the ground were simulated via an explicit dynamic analysis. An explicit FE analysis was performed using RADIOSS 2022 (Altair Engineering, Troy, MA, United States). The inputs to the analysis were the anatomically detailed FE model of the foot and a given initial condition, together with kinematic boundary conditions and time-varying muscle forces. By numerically integrating the equations of motion of the system, muscle-driven skeletal motion of the foot was generated. The simulation yielded time histories of bone motion, ligament and PA strain and tension, bone and soft tissue internal stresses, and joint and ground reaction forces. The FUJITSU Supercomputer PRIMEHPC FX1000/Server PRIMERGY GX2570 M6 (Wisteria-O/A) at the Information Technology Center, the University of Tokyo, was used in this study. The time step was set to 1 ms to ensure numerical stability and prevent oscillations.

### Comparisons with cadaveric experiment

The human foot model was validated against a cadaveric study investigating the innate mobility and mechanical properties derived from the anatomy and morphology of the human foot under axial loading [34]. In that experiment, human cadaver feet were subjected to vertical compression without tendon traction, and the resulting three-dimensional movements of the foot bones and tibia were quantified using biplane fluoroscopy [see Figure 1 in Negishi et al. [34]]. The study protocol was approved by the Office for Life Science Research Ethics and Safety at the University of Tokyo (Approval No. 19-295). This study was not a clinical trial, and therefore no clinical trial registration number is available.

In this experiment, the proximal ends of the tibia and fibula were embedded in a custom-fabricated socket made from a rubber-like polymer and attached to a shaft constrained to translate and rotate only along the vertical axis via a linear guide. We replicated this setup virtually using our developed human foot model. Specifically, the proximal ends of the tibia and fibula were embedded in a rectangular solid made of rubber material (70 mm × 50 mm × 20 mm; Young’s modulus = 4.0 MPa; Poisson’s ratio = 0.475; density = 0.0012 g/mm^3^), which was then fixed to a vertical shaft at the midpoint between the tibia and fibula. The rectangular solid, like the experimental setup, was permitted to move only along and rotate about the vertical axis.

The zero-loading condition was defined as the posture when only the vertical shaft (weighing 3.3 kg) was attached to the model. To reproduce the experimental setup more accurately and avoid introducing unnatural shear forces on the plantar surface, the foot was initially placed heel-first, with the forefoot slightly elevated, before achieving a fully plantigrade posture. This initial positioning was simulated by applying upward forces at the metatarsal heads while axially loading the tibia and fibula. This step was critical to ensure reproducibility, as the initial posture may influence foot mobility under load. Subsequently, axial loading was applied from the zero-loading condition up to 588 N (equivalent to 60 kg), and the resulting 3D translations and rotations of the foot bones were computed for comparison with the cadaveric results.

To quantify 3D bone kinematics under axial loading, a bone-fixed local coordinate system was established. At the initial position, the three orthogonal axes (x, y, z) of each bone’s local system were aligned with the global axes (X, Y, Z). The x-, y-, and z-axes corresponded to anterior, medial, and superior directions, respectively. The origin of each bone’s local coordinate system was set at its centroid. Changes in bone position and orientation due to axial loading were described using y–x–z Euler angles, following the method of Ito et al. [20]. These Euler angles captured rotations around the y-, x-, and z-axes, corresponding to plantarflexion–dorsiflexion, inversion–eversion, and internal–external rotation, respectively. Bone-to-bone joint angles for the subtalar, talonavicular, and calcaneocuboid joints were calculated as the motion of the distal bone relative to the proximal bone.

### Static simulation of quiet standing

Physiologically realistic loading conditions of the human foot during quiet standing were simulated. Assuming a body mass of 72 kg (the body weight of the CT-scanned subject), a vertical ground reaction force of approximately 353 N was applied to each foot during balanced standing. In addition, based on a structural analysis of the foot during quiet standing [9], the force generated by the triceps surae muscle (via the Achilles tendon) was estimated to be about 50% of the ground reaction force. Therefore, in this study, a downward force of 530 N and an upward force of 177 N were applied to the tibia and the calcaneal tuberosity, respectively. Dynamic calculations were performed as follows: first, an axial force of 353 N was applied to the foot from the initial position without tendon traction, and a converged static solution was obtained. Then, an additional 177 N of axial force was applied to the tibia, along with an upward force at the calcaneal tuberosity, to achieve the final static solution. To validate the human model under quiet standing conditions, we compared the simulated plantar pressure distribution with experimental measurements. The experimental data were obtained from the same subject who underwent CT scanning, using a tactile sensor system (BIG-MAT1300, Nitta, Tokyo, Japan) during balanced standing [22].

### Explicit dynamic simulation of walking

To replicate the dynamic interaction between the foot and the ground during human bipedal walking using the constructed FE model, lower leg kinematics, and ground reaction forces (GRFs) were measured from the same subject who underwent CT scanning using a motion capture system (MAC3D; Motion Analysis Corporation, Santa Rosa, CA, USA) and a force plate (EFP-S-1.5KNSA13; Kyowa Dengyo, Tokyo, Japan), respectively. The tibia and fibula in the FE foot model were driven by these kinematic data to ensure anatomical consistency in the simulation. Specifically, the motions of markers placed on the medial malleolus, lateral malleolus, and tibial tuberosity were recorded using a motion capture system. After low-pass filtering at a cutoff frequency of 20 Hz, the positions of these three landmarks in the FEM model were matched to the corresponding marker positions measured during walking by minimizing the positional error, and the resulting displacements of the three landmarks were applied to the FEM model as prescribed displacements to drive the explicit dynamic simulation.

To obtain kinematically and dynamically consistent boundary conditions, we first reproduced the experimentally observed foot posture and three components of the GRF at 20% of the stance phase by applying external forces to the tibia and fibula, together with muscle forces from the triceps surae, tibialis anterior, extensor digitorum longus, and extensor hallucis longus obtained using inverse dynamics [38]. These muscles are illustrated in Figure 1d, and the estimated muscle forces are shown in Figure 1e. This phase was selected because the tibia is nearly vertical, the plantar surface is fully in contact with the ground, and its angular velocity is relatively low [3, 4, 37]. Starting from this reference state, the experimentally measured tibial displacement and muscle forces were integrated backward in time from 20% stance to estimate the foot posture and velocity at heel contact. The foot velocity at heel contact was then reversed and used as the initial velocity for a forward dynamic simulation, in which the original tibial displacement data were applied forward in time to reproduce the walking motion.

To further reduce discrepancies between simulated and experimental GRFs, the prescribed tibial displacements were refined. For this, the differences between the simulated and experimental GRF components were computed over the stance phase and approximated by eighth-order polynomial functions using the least-squares method. These functions were scaled by empirically determined coefficients to convert GRF errors into time-dependent displacement corrections, which were added to the original tibial displacement data before rerunning the simulation. This refinement procedure yielded simulated GRFs that more closely resembled the experimental waveforms (See Supplementary Information).

Three-dimensional bone kinematics during walking were quantified by constructing bone-fixed local coordinate systems, following the approaches of Gutekunst et al. [19] and Negishi et al. [34]. Triaxial joint rotations of the talocrural (TC), talonavicular (TN), and calcaneocuboid (CC) joints, as well as the angle between the navicular and first metatarsal (N-1MT), were calculated using y–x–z Euler angles, corresponding to plantarflexion–dorsiflexion, inversion–eversion, and adduction–abduction, respectively. These joint angles were then compared with experimental kinematic data obtained during the stance phase of walking, estimated using a neural network model that tracked foot bone motion based on 41 skin-mounted markers [27], because foot bone kinematics cannot be directly measured with conventional optical motion capture. We also compared the simulated plantar pressure distribution with experimental measurements using the tactile sensor system. Furthermore, tensile force in the PA was quantified and compared with the PA force profile estimated by Caravaggi et al. [5, 6] and that measured by Erdemir et al. [15] in cadaver feet while simulating bipedal walking using a robotic gait simulator.

For the stress analysis, von Mises stress in the bony and soft tissue elements was computed for all elements at each time step. The time-varying stress distributions were then obtained by mapping these values onto the element surfaces for visualization.

## Results

### Comparisons with cadaveric experiment

**Fig. 2:**
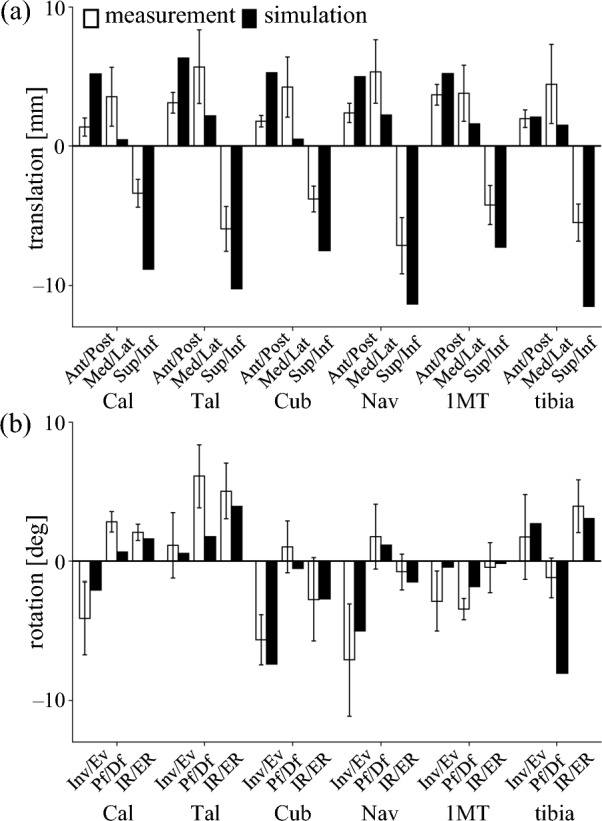
Comparison of simulated foot bone movements under axial loading with experimental data from Negishi et al. [34]. (**a**) Linear displacements along the anteroposterior, mediolateral, and vertical (superoinferior) axes. Positive values indicate anterior, medial, and superior translation. (**b**) Rotations within the coronal, sagittal, and transverse planes. Positive values represent inversion, plantarflexion, and internal rotation. Black box: simulation results; White box: experimental data. Abbreviations: Cal = calcaneus; Tal = talus; Cub = cuboid; Nav = navicular; 1MT = first metatarsal

We compared the translational and rotational responses of foot bones under axial loading in the FE simulation with experimental results from cadaveric feet reported by Negishi et al. [34] (Figure 2). Anterior and inferior translations tended to be slightly greater, while medial displacements were somewhat smaller in the simulation than in the experimental data (Figure 2a). The mean absolute differences (± standard deviations) in translation between the simulation and experimental data were 2.5 ± 1.4 mm (anteroposterior), 3.1 ± 0.5 mm (mediolateral), and 4.5 ± 1.1 mm (superoinferior). The directions of bone rotations were consistent between the simulated and measured results (Figure 2b). Corresponding angular discrepancies for inversion–eversion, plantarflexion–dorsiflexion, and internal–external rotation were 1.6 ± 0.7°, 2.9 ± 2.3°, and 0.6 ± 0.4°, respectively. Considering that the FE model was not based on the same specimens used in the experimental study, these differences are relatively minor.

### Static simulation of quiet standing

**Fig. 3:**
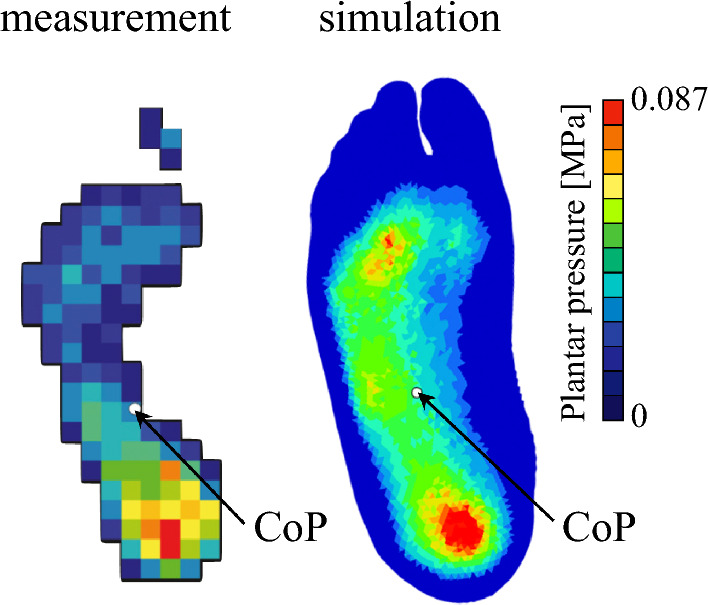
Simulated plantar pressure distribution during quiet standing. The center of pressure (COP) is marked by a white circle. Experimentally measured plantar pressure during quiet standing in a human is shown for comparison

We compared the simulated plantar pressure distribution during quiet standing with experimental data. The simulated pressure pattern and center of pressure location were largely in agreement with the measured values, while minor discrepancies existed (Figure 3). The FE model provides nodal/element-wise contact pressures at a relatively high spatial resolution, whereas the sensor outputs forces averaged over relatively large sensing elements. Consequently, highly localized pressure concentrations predicted by the FE model can be spatially averaged and attenuated in the experimental data, so that they do not appear as distinct peaks in the sensor maps, making a direct quantitative comparison between the FE-predicted and sensor-measured pressure distributions difficult.

### Explicit dynamic simulation of walking

**Fig. 4:**
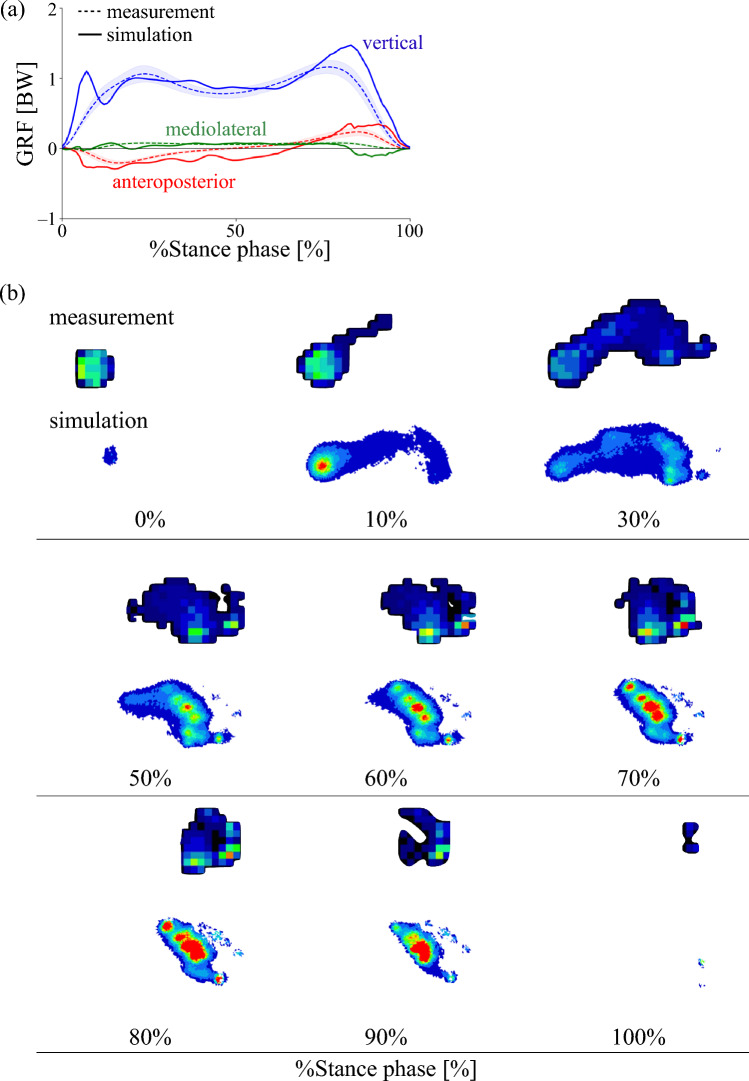
Comparison of simulated and measured ground reaction force (GRF) profiles [29] (**a**) and plantar pressure distributions (**b**) during walking. Solid lines represent simulation results; dashed lines represent experimental data. Red = anteroposterior GRF; Green = mediolateral GRF; Blue = vertical GRF. Positive values indicate posterior, medial, and superior directions

In the explicit dynamic simulation of walking, the three components of the simulated ground reaction forces during the time-normalized stance phase were compared with experimental data (mean ± standard deviation) obtained from 10 healthy adults walking at a self-selected speed [29] (Figure 4a). The mean absolute differences (± standard deviations) between the simulation and experimental data were 9.5 ± 5.5 %BW in the anteroposterior direction, 3.4 ± 3.7 %BW in the mediolateral direction, and 14.6 ± 15.1 %BW in the vertical direction. These values indicate moderate quantitative discrepancies, particularly in the vertical component; however, key biomechanical characteristics were well reproduced. The anterior–posterior ground reaction force exhibited a braking phase in early stance and a propulsive phase in late stance, with amplitudes closely matching the measured data. The mediolateral component showed a noticeable lateral peak toward the end of stance, which may warrant further investigation, but the overall pattern was largely consistent. The vertical component marginally reproduced the characteristic double-peaked profile observed in human walking. The time-varying plantar pressure distribution obtained from the simulation was compared with the experimentally measured distribution at 10% intervals throughout the stance phase (Figure 4b); however, the results at 20% and 40% were similar to that at 30%, and thus are not shown. Although the patterns were not identical, partly due to the fact that highly localized pressure concentrations predicted by the FE model can be spatially averaged and attenuated in the experimental data, the center of pressure progressed from the heel to the toes and the overall footprint shape and pressure distribution were generally similar, indicating that the simulated pattern is broadly consistent with the measured data.

**Fig. 5:**
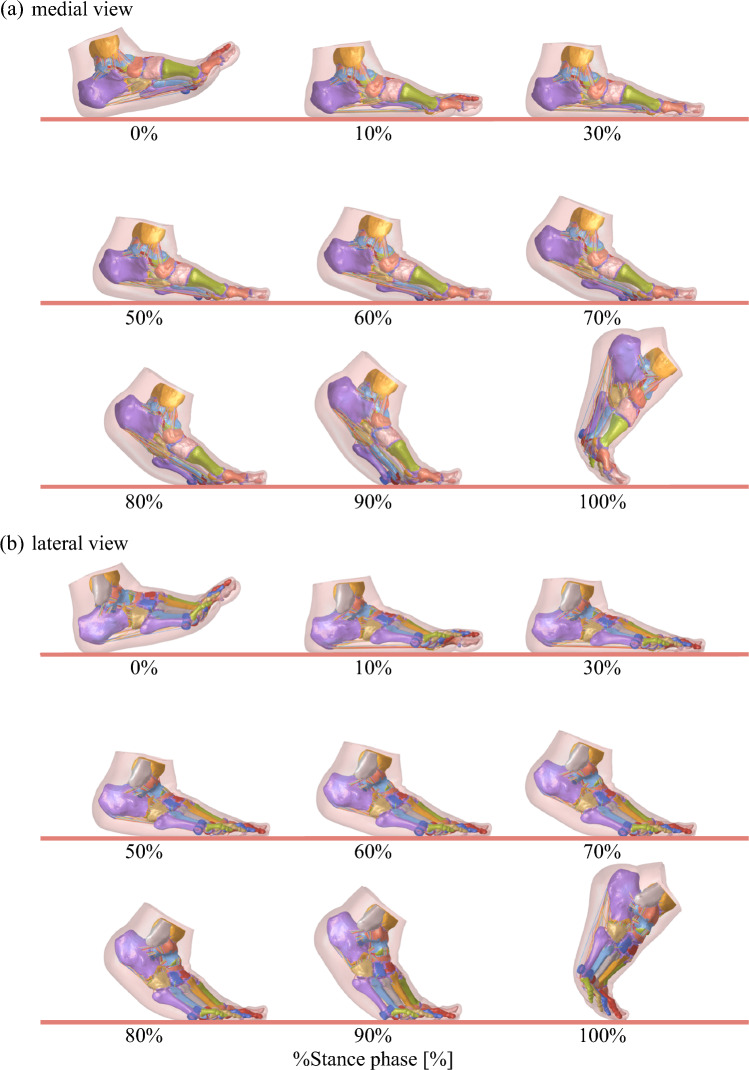
Simulated foot movements during walking. (**a**) Medial and (**b**) lateral views. The medial view is mirrored to match the left–right progression of time

**Fig. 6:**
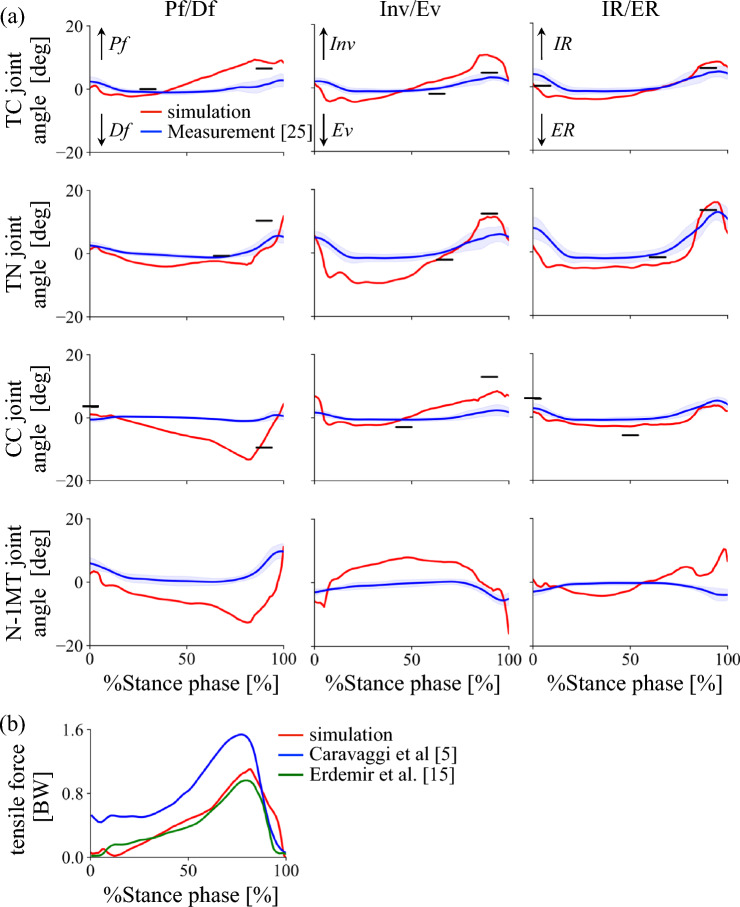
Comparison of simulated joint angles (**a**) and plantar aponeurosis force profiles (**b**) during walking. Simulated joint angles include the talocalcaneal (TC), talonavicular (TN), and calcaneocuboid (CC) joints, as well as the rotational angle of the first metatarsal relative to the navicular (N–1MT). Red lines: simulation; Blue lines: experimental data from Matsumoto et al. [25] and Caravaggi et al. [5]. Green line: experimental data from Erdemir et al. [15]. Horizontal bars indicate the mean peak angles and their timing as measured using biplanar fluoroscopy based on published data (Koo et al. [23]; Phan et al. [38])

The simulated foot movements during the stance phase were presented in Figure 5 (See Supplementary Information for a video). The changes in joint angles and their comparisons with the experimentally obtained corresponding joint angles [27] were presented in Figure 6a. The mean absolute differences (± standard deviations) between the simulation and experimental kinematics were as follows: 3.0 ± 1.9°, 3.7 ± 2.6°, and 2.1 ± 1.4° for the TC joint; 4.9 ± 2.9°, 2.3 ± 1.0°, and 4.0 ± 1.8° for the TN joint; 2.9 ± 1.7°, 5.0 ± 3.5°, and 1.9 ± 0.6° for the CC joint; and 5.4 ± 2.1°, 7.5 ± 3.5°, and 3.6 ± 2.9° for the N–1MT angle, for inversion/eversion, plantarflexion/dorsiflexion, and internal/external rotation, respectively. Although there are some discrepancies, the changes in foot joint angles in the simulation are generally consistent with the experimental kinematic data. In addition, the maximum and minimum values of the estimated joint angle profiles and the timing of these peaks generally aligned with those reported in previous studies using biplanar fluoroscopy [25, 41], indicating that our simulation is also largely consistent with those measured data.

The simulated tension profile of the PA during walking (Figure 6b) showed a pattern of increasing force throughout most of the stance phase, consistent with the findings of Caravaggi et al. [5, 6], who reported that the tensile force of the PA reached approximately 1.5 times body weight. In our simulation, the peak tensile force reached approximately 1.1 times body weight, showing a similar waveform, but the peak magnitude was smaller. However, the simulated tension profile was more similar to that reported by Erdemir et al. [15]. The mean absolute difference (± standard deviation) between the simulation and experimental tensile force profile was 19 ± 17 %BW, indicating generally good agreement with Erdemir et al. [15].

**Fig. 7:**
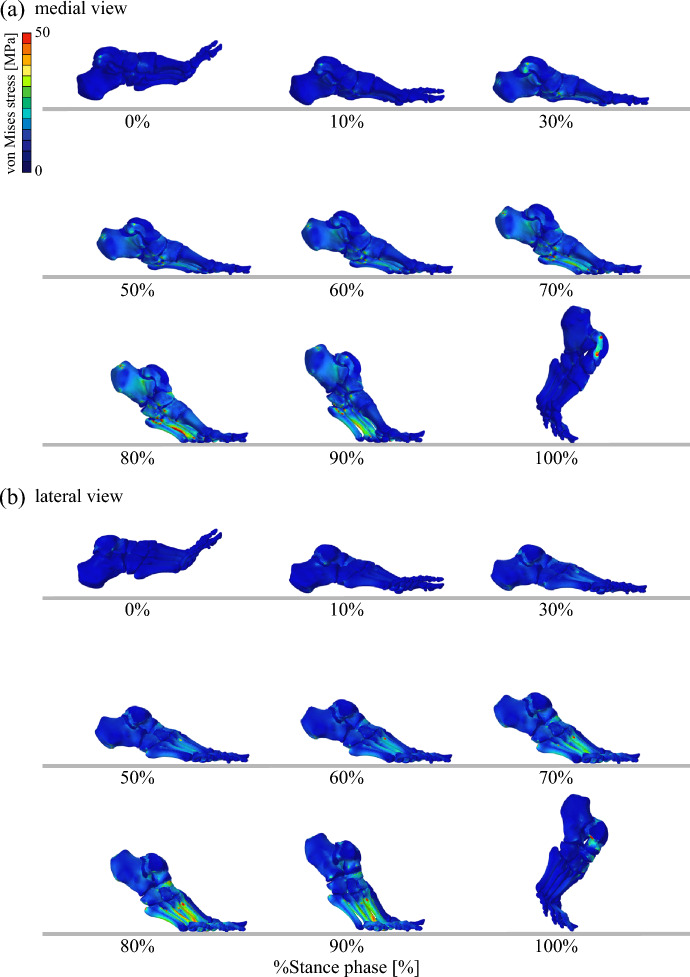
Simulated von Mises stress distributions of the foot bones during walking. (**a**) Medial and (**b**) lateral views. The medial view is mirrored to match the left–right progression of time

**Fig. 8:**
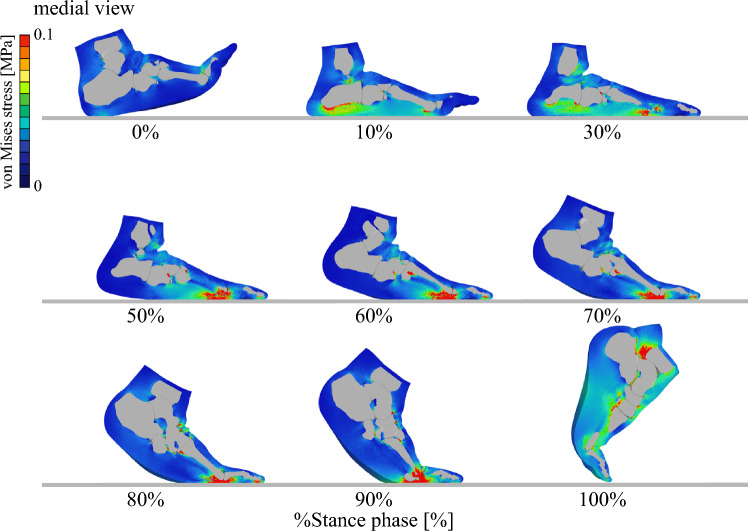
Simulated von Mises stress distributions of the soft tissue during walking

Figures 7 and 8 illustrate the time-varying von Mises stress distribution in the bones and soft tissues, respectively, during walking (See Supplementary Information for a video). Although such stress changes cannot be measured directly in vivo, the simulation enables their visualization. The results suggest that bone stress is concentrated in the metatarsals, while soft tissue stress is primarily distributed under the plantar regions of the calcaneus and metatarsophalangeal joints.

## Discussion

This study demonstrated that the explicit finite element simulation of the foot, driven by experimentally measured tibial kinematics and estimated muscle forces, successfully replicated key mechanical behaviors of the foot as it interacts with the ground during walking. The simulation results were generally consistent with experimental data in terms of kinematics, ground reaction forces, and plantar pressure distribution throughout the stance phase. Considering that the model foot and the foot used for the kinematic measurements (Figures 2 and 6) were not identical, perfect agreement could not be expected; nevertheless, the correspondence observed was generally consistent. This was achieved by refining the prescribed tibial displacements after the initial calculation. Because of experimental and modeling errors, direct use of the motion capture data as input did not provide satisfactory results in the initial simulation. We therefore adjusted the tibial displacements based on the differences between the simulated and experimental GRF waveforms, which led to a much closer match (Supplementary Information). The reproducibility of the simulations primarily depends on the availability of the measured kinematic and kinetic data and the refinement algorithm, both of which are explicitly described in the present study.

This level of agreement indicates that the model is sufficiently accurate for qualitative analysis and hypothesis testing, although refinements in geometry and subject-specific material properties would be necessary for precise quantitative predictions. The model appears to capture the essential aspects of foot mechanics during walking. Although some discrepancies remain, likely due to simplifications in the modeling of soft tissues, ligaments, and contact properties, the present framework provides a useful basis for investigating the relationship between form and function in the musculoskeletal system and for advancing our understanding of the pathogenesis of foot and ankle disorders.

Previous studies performed 3D FE analyses of the human foot during walking [1, 33, 55]. However, these 3D FE foot models have typically been validated primarily against experimentally measured plantar pressure distributions, with limited consideration of horizontal ground reaction forces and bone kinematics. Consequently, while they successfully reproduced overall foot mechanics, their ability to accurately capture foot dynamics, particularly in terms of 3D joint motion and shear loading, has remained uncertain. In contrast, the present model was validated not only against plantar pressure but also against full three-component ground reaction forces and foot bone and joint kinematics, providing a clearer evaluation of its dynamic behavior. Furthermore, no previous studies combined an anatomically detailed 3D FE representation of the human foot with a continuous simulation of the entire stance phase, from heel contact to toe-off, under muscle-driven loading conditions. To our knowledge, the present framework is the first to achieve this combination.

By enabling the estimation of internal forces, stresses, and strains within foot structures during gait, parameters that are not directly measurable in experimental settings, this model offers valuable insights into the biomechanics of bones, soft tissues, ligaments, and joints during human walking. Although the present results require further validation for quantitative accuracy, they show good qualitative agreement with known biomechanical patterns. Such information is essential for understanding the development of foot pathologies, including plantar fasciitis [29, 30], hallux valgus [36], ankle osteoarthritis [45, 46], and diabetic foot ulcers [21]. Moreover, this modeling framework can be extended to assess the mechanical effects of footwear design, orthotic interventions, and surgical procedures.

The adoption of an explicit dynamic simulation approach proved advantageous for handling complex contact interactions and large deformations within the foot during walking. It must be noted, however, that the method required substantial computational time. In the present study, the calculation of foot dynamics from foot contact to toe-off (Figure 5) required approximately 14 hours using the supercomputer. While such computational times are acceptable for research purposes, clinical or design applications would require a significant reduction in runtime through model optimization, parallel computing, or reduced-order modeling techniques. Balancing computational efficiency with anatomical and biomechanical realism remains a challenge for applying such simulations to the detailed understanding of musculoskeletal dynamics and their medical applications.

This study has several limitations. First, the foot model was constructed based on CT data from a single individual and therefore does not capture the natural variation in foot morphology. Second, the material properties used in the finite element analysis were compiled from various literature sources. As a result, parameters such as ligament cross-sectional areas were not individually scaled due to insufficient data for accurate adjustment. Additionally, the foot’s soft tissues were simplified as a single homogeneous material, whereas in reality, they consist of structurally and mechanically distinct components, including fat, muscle, and tendons. Although the soft tissue material parameter was derived from experimental measurements of the heel pad as a whole rather than from individual tissue components, this level of simplification is typical in foot finite element models. Nevertheless, the anatomical differences between the modeled and actual foot likely contributed to discrepancies in the simulation results. A further limitation is that the time histories of muscle forces were obtained by digitizing previously published curves and prescribing them using linear interpolation. Although this approximation is unlikely to affect the dynamic behavior of the model, using smoother, more accurate muscle force profiles would likely provide a more realistic representation of the loading conditions. Finally, we did not perform a systematic sensitivity analysis (e.g., mesh density, material parameters) because of the high computational cost of the explicit simulations. Future work should address this to further evaluate the robustness of the model. Despite these limitations, however, we believe that the present simulation successfully captured key aspects of foot mechanics and offers meaningful insights into foot function, highlighting the potential of this modeling approach for future research in comparative morphology, biomechanics, and clinical applications. Therefore, the present model provides a framework that can be used to gain deeper insight into the mechanical principles underlying foot function in both evolutionary and clinical contexts.

## Supplementary Information

Below is the link to the electronic supplementary material.

Supplementary file1 (MP4 24760 kb)

Supplementary file2 (XLSX 14 KB)

Supplementary file3 (DOCX 363 KB)

## Data Availability

The datasets generated and/or analyzed during the current study are available from the corresponding author upon reasonable request.
