# Supplementary material for: Simulating human foot mechanics during walking based on an anatomically detailed forward dynamic finite element model
Source: Ann Biomed Eng. 2026 Jan 13;54(5):1435–49. doi: 10.1007/s10439-026-03984-3 (PMC13091900; doi:10.1007/s10439-026-03984-3)
Supplement: Supplementary file 3 — Supplementary file3 (DOCX 363 KB) [file 10439_2026_3984_MOESM3_ESM.docx]

**Simulating human foot mechanics during walking based on an anatomically detailed forward dynamic finite element model**

Kohta Ito^1,2,†^, Yuka Matsumoto^1,3,†^, Hiroyuki Seki^4,5^, Takeo Nagura^5^, and Naomichi Ogihara^1^

1. Department of Biological Science, Graduate School of Science, The University of Tokyo, Tokyo, Japan
2. Graduate School of Human Sciences, The University of Osaka, Suita, Japan
3. Research Development Center, Saitama Prefectural University, Koshigaya, Japan
4. Department of Orthopedic Surgery, Tachikawa Hospital, Tachikawa, Japan
5. Department of Clinical Biomechanics, Keio University School of Medicine, Tokyo, Japan

†) These authors have contributed equally to this work and share first authorship.

Corresponding Author

Naomichi Ogihara

Department of Biological Science, Graduate School of Science,

The University of Tokyo,

7-3-1 Hongo, Bukyo-ku, Tokyo 113-0033, Japan

e-mail: [ogihara@bs.s.u-tokyo.ac.jp](mailto:ogihara@bs.s.u-tokyo.ac.jp)

**Supplementary material**

**Supplementary table**

Cross-sectional areas of the ligaments and corresponding references (cross-sectional area.xlsx).

**Supplementary video**

Simulated temporal changes in foot movements and von Mises stress distributions of the foot bones and soft tissue during walking (FEM_walking.mp4). The video sequentially presents the simulated foot motion, the von Mises stress distributions of the foot bones (shown from medial and lateral views, respectively), and the von Mises stress distribution within the soft tissue. The medial view is mirrored to match the left-to-right temporal progression of the walking cycle.

**Supplementary figure**

Figure S1. Differences in the GRF waveforms before and after refinement of the boundary conditions (a). The motion-capture trajectories of the markers placed on the lateral malleolus, medial malleolus, and tibial tuberosity were adjusted (refined) to obtain the final simulation result (b).
